# Efficient Decolorization and Preparation of *Sparassis crispa* Polysaccharides Using Amino-Modified Silica Gel and Evaluation of Their Biological Activites

**DOI:** 10.3390/foods14244214

**Published:** 2025-12-08

**Authors:** Jiebo Chen, Chunyan Zhang, Cheng Peng, Lu Wang, Shoujing Zheng

**Affiliations:** 1 National Engineering Research Center of Sugarcane, College of Food Science, Fujian Agriculture and Forestry University, Fuzhou 350002, China; zcy_chunyanz@163.com (C.Z.); zzpcnb@163.com (C.P.); wanglu@fafu.edu.cn (L.W.); 2 Jinshan College, Fujian Agriculture and Forestry University, Fuzhou 350002, China; zsj_fafu@163.com

**Keywords:** *Sparassis crispa* polysaccharides, amino-modified silica gel adsorbent, decolorization

## Abstract

This study synthesized an efficient amino-modified silica gel decolorizer by aminating silica hydroxyls, characterized via SEM, FT-IR, N_2_ adsorption and XPS. It investigated its decolorization of *Sparassis crispa* crude polysaccharides using decolorization rate and polysaccharide retention as indices, revealing pigment adsorption mechanisms. Polysaccharide activity preservation by physical adsorption was evaluated via antioxidant (DPPH, ABTS, •OH scavenging) and zebrafish caudal fin repair assays. The results showed 79.85% decolorization and 86.54% polysaccharide retention within 5 min, with over 75% decolorization after five cycles of reuse. Results suggest that pigment adsorption may involve interactions between amino groups and chromophoric compounds. The decolorized polysaccharides showed better antioxidant and zebrafish caudal fin repair activities than those treated with traditional H_2_O_2_. These findings support the development of efficient, low-damage decolorization strategies for edible and medicinal polysaccharides.

## 1. Introduction

*Sparassis crispa* (*S. crispa*) is a valuable edible and medicinal fungus rich in bioactive polysaccharides, mainly β-glucan, which accounts for 40–43% of its dry weight [1]. Studies have shown that β-glucan from *S. crispa* possesses a variety of biological activities, including anti-tumor, immunomodulatory, hematopoiesis-enhancing, wound-healing, antioxidant, and antibacterial effects [2,3,4,5]. As a significant class of biological macromolecules, polysaccharides have broad application potential in the fields of food, medicine, and cosmetics. High-purity polysaccharides are considered essential for exploring structure–activity relationships and enabling practical applications. However, crude polysaccharides are often accompanied by pigment impurities, which not only hinder investigations of their structure and biological activities but may also exert adverse effects on their functionality [6,7,8].

Therefore, decolorization is a critical step in the purification of polysaccharides. Current decolorization methods are mainly classified into hydrogen peroxide (H_2_O_2_) oxidative chemical methods and physical adsorption-based approaches. Ren et al. [9], Chen et al. [10] and Shao et al. [11] used H_2_O_2_ to decolorize crude polysaccharides extracted from *Rehmannia glutinosa*, *Sargassum*, and *Astragalus membranaceus*, respectively. However, H_2_O_2_ tends to damage polysaccharide structures and generate residues. Adsorption-based decolorization is a physical approach that utilizes adsorbents with high specific surface area and porous structures, such as activated carbon and macroporous resins. Activated carbon demonstrates rapid pigment adsorption but lacks selectivity, often leading to significant polysaccharide loss. Furthermore, activated carbon is difficult to separate from polysaccharides, compromising product quality [12,13]. Macroporous resins are also widely used for polysaccharide decolorization. For instance, Shun et al. [14] applied D301 macroporous resin to decolorize water-soluble crude polysaccharides from *Lepista nuda*, achieving a decolorization rate exceeding 85% within 2.5 h, but with a polysaccharide retention rate below 56%. Shi et al. [15] employed an anion exchange macroporous resin (AEMR) to decolorize crude polysaccharides from *Toona sinensis* buds (PSTS), reaching a decolorization rate above 90% at 240 min, while the polysaccharide recovery rate dropped below 50%. Hu et al. [7] optimized decolorization conditions for crude polysaccharides from *Rhododendron* leaves using macroporous resin adsorption via Analytic Hierarchy Process (AHP) and Response Surface Methodology (RSM), and found that when the decolorization time exceeded 70 min, the decolorization rate was less than 72.26%, and the recovery rate was below 81.56%. Although macroporous resins show promising decolorization performance, their long adsorption equilibrium time results in low efficiency, which limits industrial applicability.

Silica gel, featuring high specific surface area, stable mechanical properties, and tunable particle and pore sizes, presents potential as an alternative adsorbent. Moreover, its surface is rich in hydroxyl groups, allowing for easy chemical modification into functional adsorbent materials. The major pigment impurities in natural polysaccharide extracts are polyphenols and their derivatives, whose aromatic structures can form strong π–π interactions with amino groups [16,17,18,19]. Although amino-modified silica materials have been previously explored, their application to the decolorization of *S. crispa* polysaccharides has not been systematically evaluated. Thus, the present study aimed to develop an efficient physical decolorant for crude polysaccharides from *S. crispa* by modifying silica gel with amino groups. The decolorization performance is evaluated in terms of decolorization rate and polysaccharide retention rate. The structure and properties of the modified silica gel were characterized using Scanning Electron Microscopy (SEM), Fourier Transform Infrared Spectroscopy (FTIR), Brunauer–Emmett–Teller (BET) analysis, and X-ray Photoelectron Spectroscopy (XPS) to elucidate the adsorption mechanism. Furthermore, the antioxidant activity and zebrafish caudal fin regeneration were used to compare the biological activities of *S. crispa* polysaccharides obtained via physical versus H_2_O_2_ decolorization. This study aimed to synthesize and characterize amino-modified silica, evaluate its decolorization efficiency and biological effects in comparison with H_2_O_2_, and offer valuable insights for the efficient extraction and utilization of *S. crispa* polysaccharides.

## 2. Materials and Methods

### 2.1. Materials, Reagents and Experimental Animals

*S. crispa* were purchased from Rongyi Edible Fungi Technology Co., Ltd. (Fuzhou, China). 3-(2-Aminoethylamino) propyltriethoxysilane (APS, 99%) was purchased from Aladdin Biochemical Technology Co., Ltd. (Shanghai, China). Silica gel (SiO_2_, 5 µm, 100 Å) was purchased from Zhongpu Technology Co., Ltd. (Fuzhou, China). All the chemicals were of analytical reagent grade unless otherwise stated. Zebrafish embryos of AB wild-type and lyz fluorescent strains were provided by Yixiyue Biotechnology Co., Ltd. (Weifang, China).

### 2.2. Preparation of Amino-Modified Silica Gel

20 g of silica gel was placed into a 150 mL three-neck round-bottom flask, followed by 50 mL of water-free toluene and different volumes of APS (0.85, 1.7, 2.05, or 2.4 mL). The mixture was stirred under nitrogen at 50 °C for 24 h under reflux. After cooling and filtering, the product was washed with 25 mL of toluene and methanol (3 × 25 mL) and then vacuum-dried at 80 °C overnight to obtain amino-modified silica gels with different grafting levels. Based on the APS used, these modified silica gels were labeled as PSA-1 (0.85 mL APS), PSA-2 (1.7 mL APS), PSA-3 (2.05 mL APS), and PSA-4 (2.4 mL APS).

### 2.3. Structural Characterization of Silica Gel

#### 2.3.1. Fourier-Transform Infrared Spectroscopy (FTIR)

FTIR spectra of silica gel and the amino-modified silica gel powders were recorded on a Nicolet iN 10MX (Thermo Fisher, Waltham, MA, USA). Each sample was ground with KBr (1:100), pressed into pellets, and analyzed with OMNIC9.0 software.

#### 2.3.2. Scanning Electron Microscopy (SEM)

Surface morphology was analyzed using a Hitachi S-4800 SEM (Tokyo, Japan) at 10 kV and 7 mA.

#### 2.3.3. N_2_ Adsorption–Desorption Analysis

Specific surface area, pore volume, and pore size distribution were measured with a Kubo × 1000 analyzer (Biaode, Beijing, China). Samples were pretreated at 573 K under vacuum and tested at −197 °C in liquid nitrogen. BET analysis was used for surface area (P/P_0_ = 0.05–0.35), and BJH analysis for pore distribution from the desorption branch. Pore volume was taken at P/P_0_ = 0.995.

#### 2.3.4. X-Ray Photoelectron Spectroscopy (XPS)

XPS analysis was performed using an APOLLOX spectrometer with Al Kα radiation (22.8 W). The C1s peak at 284.8 eV was used for calibration, and avantage software was used for peak fitting.

### 2.4. Extraction of Crude Polysaccharides from S. crispa

A total of 25 g of *S. crispa* powder was mixed with 500 mL of distilled water and stirred at 60 °C for 3 h. The mixture was centrifuged at 8000 rpm for 10 min to remove residues. The supernatant was collected, mixed with 2% (*w*/*v*) diatomaceous earth, and filtered under vacuum. The filtration was passed through a 10 KDa hollow fiber membrane to remove small molecules and concentrated four-fold. The crude polysaccharide solution was stored at −18 °C for subsequent decolorization experiments.

### 2.5. Adsorption of Pigments from Crude Polysaccharides by Silica Gel

Silica gel and the amino-modified silica gel were added separately into crude polysaccharide solutions. The mixtures were stirred at room temperature for 5 min and centrifuged at 8000 rpm for 10 min. The absorbance at 420 nm was measured before and after decolorization to calculate the decolorization rate using Equation (1):

(1) $$\mathrm{Decolorization}\;\mathrm{rate}\;(\%)\;=\;\left[(A_{0}-A_{e}\right)/A_{0}]\times 100$$

where *A*_0_ and *A_e_* are the absorbance at 420 nm before and after decolorization, respectively.

Polysaccharide content was determined using the phenol-sulfuric acid method at 490 nm, with glucose as the standard [20]. Each sample was measured in triplicate. The polysaccharide retention rate was calculated using Equation (2):

(2) $$\mathrm{Retention}\;\mathrm{rate}\;(\%)\;=\;(C_{t}/C_{0})\times 100$$

where *C*_0_ and *C_t_* represent polysaccharide concentrations before and after decolorization, respectively.

After adsorption decolorization of the crude polysaccharide solution with PSA, proteins were removed using the Sevage reagent. Four volumes of anhydrous ethanol were added, and the mixture was incubated at 4 °C overnight. The precipitate was collected by centrifugation and subsequently freeze-dried to obtain *S. crispa* polysaccharides powder for further bioactivity assays.

### 2.6. Reusability and Regeneration of PSA-2

#### 2.6.1. Reusability Test

Based on the decolorization performance, PSA-2 was selected for reuse evaluation. A total of 0.15 g of PSA-2 was added to 50 mL of crude polysaccharide solution, stirred at 420 rpm for 5 min, and centrifuged (8000 rpm, 5 min). The separated PSA-2 was reused directly without washing, and the decolorization and retention rates were determined. This cycle was repeated five times.

#### 2.6.2. Regeneration Test

The method was adapted from Kang et al. [21] with appropriate modifications. For regeneration, 0.15 g of PSA-2 was first used to adsorb pigment from 50 mL of crude polysaccharide solution. After separation, it was treated with 0.1 M NaOH and stirred for 120 min at 420 rpm. The regenerated PSA-2 was washed with deionized water to neutral pH, centrifuged, and freeze-dried. The treated adsorbent was then reused in the same manner for five cycles to evaluate its reusability.

### 2.7. Preparation of H_2_O_2_ Decolorized Polysaccharides

For comparison with the PSA decolorization method, the crude *S. crispa* polysaccharides were subjected to decolorization using H_2_O_2_. 6% (*w*/*v*) H_2_O_2_ solution was added to the 50 mL of the crude polysaccharide solution prepared in Step 2.4. The pH was adjusted to 8.0 with 6 M NaOH, and the mixture was shaken at 50 °C for 180 min [11]. Proteins were removed using the Sevage reagent. Thereafter, four volumes of anhydrous ethanol were added, and the mixture was incubated at 4 °C overnight. The precipitate was collected by centrifugation and subsequently freeze-dried to obtain *S. crispa* polysaccharides powder for further bioactivity assays.

### 2.8. Bioactivity Evaluation of S. crispa Polysaccharides

#### 2.8.1. In Vitro Antioxidant Activity

(1) DPPH Radical Scavenging

The polysaccharide solutions (1.0–5.0 mg/mL) treated with H_2_O_2_ or PSA-2 were mixed with 0.2 mM DPPH ethanol solution (1:1, *v*/*v*) and incubated in the dark for 30 min at room temperature. Absorbance was measured at 517 nm. Ascorbic acid (VC) was used as a positive control. Scavenging activity was calculated by Equation (3):

(3) $$\mathrm{Scavenging}\;\mathrm{rate}\;(\%)\;=\;[1-\left(A_{1}-A_{2}\right)/A_{3}]\times 100$$

where *A*_1_ is absorbance of sample+ DPPH, *A*_2_ is DPPH+ ethanol, and *A*_3_ is sample+ ethanol.

(2) ABTS Radical Scavenging

ABTS stock solution was prepared by mixing 7.0 mM ABTS and 2.45 mM potassium persulfate and incubating in the dark for 12 h. The working solution (A_734_ = 0.700 ± 0.02) was mixed with sample solutions (1.0–5.0 mg/mL), incubated for 6 min, and absorbance was measured at 734 nm. Ascorbic acid (VC) was used as a positive control. Scavenging activity was calculated by Equation (4):

(4) $$\mathrm{Scavenging}\;\mathrm{rate}\;(\%)\;=\;[1-\left(B_{1}-B_{2}\right)/B_{3}]\times 100$$

where *B*_1_ is sample + ABTS, *B*_2_ is water + ABTS, and *B*_3_ is sample + water.

(3) Hydroxyl Radical Scavenging

The polysaccharide solutions (1.0–5.0 mg/mL) were mixed with 9 mM FeSO_4_·7H_2_O, 9 mM H_2_O_2_, and 9 mM salicylic acid (1:1:1:1, *v*/*v*), reacted at 37 °C for 30 min, and the absorbance was measured at 562 nm. Ascorbic acid (VC) was used as a positive control. The scavenging rate was calculated using Equation (5):

(5) $$\mathrm{Scavenging}\;\mathrm{rate}\;(\%)\;=\;[1-\left(A_{i}-A_{j}\right)/A_{o}]\times 100$$

where *A_i_* is sample + Fe^2+^ + H_2_O_2_, *A_j_* is sample + Fe^2+^, and *A_o_* is water + Fe^2+^ + H_2_O_2_.

#### 2.8.2. Zebrafish Caudal Fin Regeneration

Healthy, non-malformed, and normally developed 48 hpf (hours post-fertilization) AB strain zebrafish embryos were selected. All animal experiments were approved by the Animal Ethics Committee of Fujian Agriculture and Forestry University (Approval No.2.8 Data Analysis PZCASFAFU25066). The 48 hpf zebrafish were placed in 15 mL of deionized water supplemented with 10 µL of anesthetic solution for anesthetic treatment. Subsequently, using the posterior segment of the ventral pigmentation gap of the caudal fin as an anatomical reference, the caudal fin was transversely transected under a stereomicroscope with a sterile blade. Immediately afterward, the embryos were transferred to clean culture water to allow them to regain vitality, then grouped into 6-well plates with 30 embryos per well. Four groups were set up: the model control group, sample group, comparison group, and positive control group. Specifically, the model control group was supplemented with 4 mL of culture solution; the sample group received 4 mL of *S. crispa* polysaccharide solution decolorized with 0.15 g PSA-2 at concentrations of 0.5 mg/mL, 1.0 mg/mL, and 1.5 mg/mL, respectively; the comparison group was added with 4 mL of *S. crispa* polysaccharide solution decolorized by H_2_O_2_ at concentrations of 0.5 mg/mL, 1.0 mg/mL, and 1.5 mg/mL, respectively; and the positive control group was added with 4 mL of culture solution containing 0.5 mg/mL *Rehmannia glutinosa* extract. All groups were incubated in a biochemical incubator at (28.5 ± 1) °C for 48 h. The zebrafish caudal fins were photographed under a stereomicroscope, and ImageJ analysis was performed, which allowed consistent fin-length measurement.

### 2.9. Data Analysis

Zebrafish images were analyzed using ImageJ 1.8.10 software with a 1.00 mm scale bar (*n* = 10 per group). All other experiments were independently replicated three times (*n* = 3). Data was processed using Excel software and plotted with Origin 2022 software. Statistical significance was analyzed by performing an ANOVA for intergroup differences using SPSS 27 software, with a *p* < 0.05 considered statistically significant.

## 3. Results

### 3.1. Structural Characterization of Silica Gel-Based Decolorizing Adsorbents

#### 3.1.1. FT-IR Analysis

**Figure 1:**
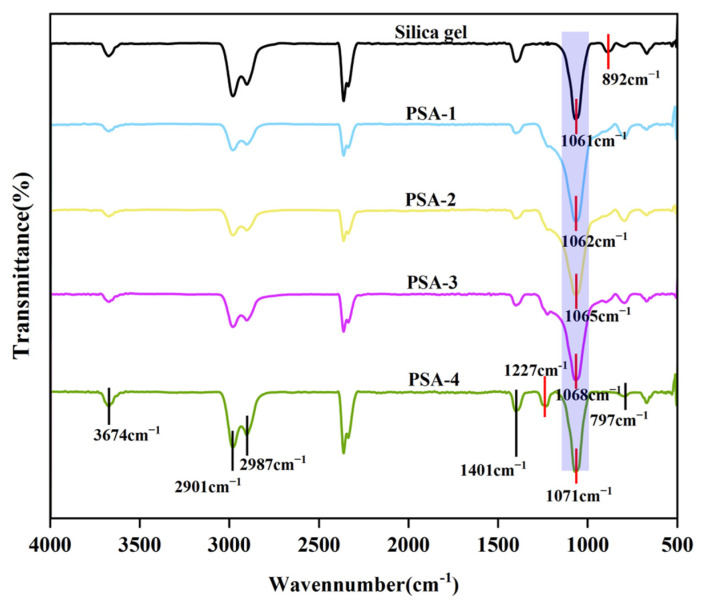
The FTIR spectra of silica gel and the amino-modified silica gel. Note: The purple color indicates that the characteristic peak at this position undergoes a blue shift.

**Figure 2:**
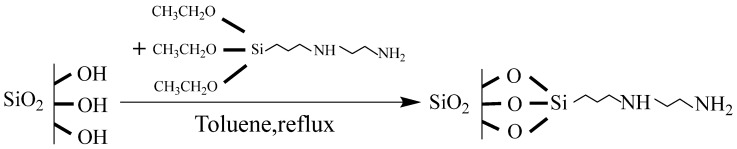
Schematic diagram of the synthesis of amino-functionalized silica gel decolorizing agent.

Fourier-transform infrared (FT-IR) spectroscopy was employed to characterize both silica gel and the amino-modified silica gel, as shown in Figure 1. The broad absorption band at 3674 cm^−1^ is attributed to the O–H stretching vibrations of surface Si–OH groups and adsorbed water [22]. The peaks at 2987 cm^−1^ and 2901 cm^−1^ correspond to the asymmetric and symmetric stretching vibrations of C–H bonds, respectively. The sharp absorption band at 1061 cm^−1^ is assigned to the Si–O–Si stretching vibration, while the peak at 1401 cm^−1^ is due to C–H bending vibration. The absorption at 1227 cm^−1^ is attributed to the C-N bending vibration. The absorption at 892 cm^−1^ is ascribed to the bending vibration of Si–OH groups [23], and the strong peak at 797 cm^−1^ corresponds to the bending vibration of Si–O bonds [24]. Comparison of the FT-IR spectra of silica gel and the amino-modified silica gel revealed that their primary characteristic peaks occurred at similar positions, indicating that the surface modification did not disrupt the fundamental silica framework. However, with increasing amounts of 3-(2-Aminoethylamino) propyltriethoxysilane (APS) used in the modification, the intensity of the characteristic peak at 1227 cm^−1^, which belongs to the C-N bending vibration, increased gradually, confirming that the APS modifier was successfully grafted onto the silica gel. In addition, the characteristic peak at 1061 cm^−1^ (Si–O–Si stretching) exhibited a blue shift, while the intensity of the 892 cm^−1^ peak (Si–OH bending) gradually decreased. Simultaneously, the 797 cm^−1^ peak (Si–O bending) became more pronounced. These changes suggest that a condensation reaction occurred between surface Si–OH groups and APS, leading to the formation of new Si–O–Si linkages and a concurrent reduction of Si–OH groups. The reaction mechanism is illustrated in Figure 2. 

#### 3.1.2. XPS Analysis

**Figure 3:**
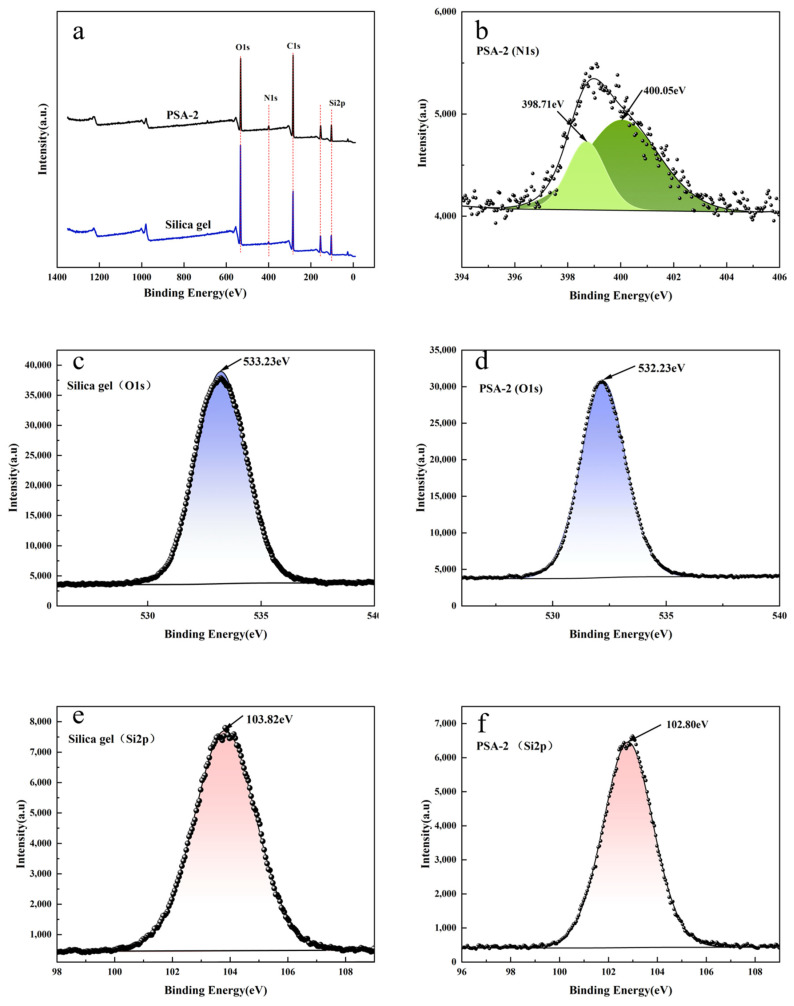
XPS spectra of silica gel and amino-modified PSA-2: (**a**) Full-scan spectra of silica gel and amino-modified silica gel; (**b**) XPS spectra of N1s for silica gel and amino-modified silica gel; (**c**,**d**) XPS spectra of O1s for silica gel and amino-modified silica gel; (**e**,**f**) XPS spectra of Si2p for silica gel and amino-modified silica gel.

To further confirm the surface amino-modified silica gel by the APS coupling reagent, X-ray photoelectron spectroscopy (XPS) was conducted on both silica gel and the PSA-2 sample. As shown in Figure 3a, the full scan spectrum of the silica gel displayed only a weak N1s signal near 399 eV, which is mainly attributed to background contamination or adsorbed nitrogen-containing species from the environment during measurement [25]. In contrast, the PSA-2 sample exhibited a pronounced N1s peak, indicating a significant presence of nitrogen atoms on the modified surface. High-resolution N1s spectrum of PSA-2 (Figure 3b) was further deconvoluted, revealing two distinct peaks at binding energies of 398.71 eV and 400.05 eV, corresponding to N1s signals from primary amine groups (-NH_2_) and secondary amine groups (-NH-), respectively [26]. These results confirm the successful introduction of both –NH_2_ and –NH– groups from the APS coupling onto the silica gel surface. Further, by comparing the binding energies of O1s and Si2p before and after modification (Figure 3c–f), it was observed that after amino modification, the Si in Si-OH groups on the silica gel surface is linked to weak electron-donating groups (alkyl and amino groups). Additionally, the O in the ethoxy group is connected to both Si and C, and its electron-withdrawing effect is weaker than that of O in -OH groups. Consequently, the electron cloud density of the grafted Si is higher, and its binding energy is lower than that of Si in surface Si-OH groups. As a result, the Si atoms grafted with silane exhibit a higher electron cloud density, leading to a reduced Si2p binding energy from 103.82 eV to 102.80 eV (Figure 3e,f). Similarly, the O atoms involved in the Si–OH groups before modification displayed a higher binding energy due to their stronger electron-withdrawing environment. After modification, the O atoms were bonded to both grafted Si and ethyl groups; due to the higher electronegativity of C relative to Si, electron withdrawal by C is less effective, leading to a higher electron density around O and thus a decrease in the O1s binding energy from 533.23 eV to 532.23 eV (Figure 3e,f). In summary, these XPS results confirm that the ethoxy groups of APS successfully underwent condensation reactions with surface Si–OH groups, resulting in effective amination of the silica gel surface [27].

#### 3.1.3. SEM Analysis

**Figure 4:**
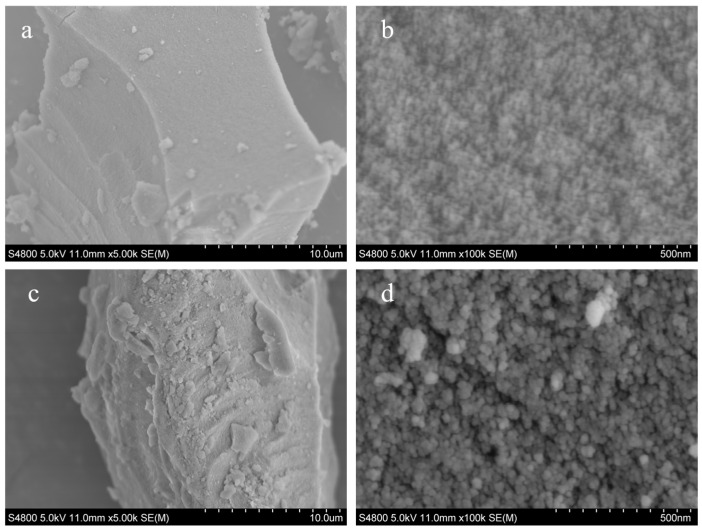
Scanning electron microscopy (SEM) images of silica gel and the amino-modified silica gel at different magnifications: (**a**,**b**) silica gel; (**c**,**d**) amino-modified silica gel PSA-2.

The surface morphologies of silica gel and amino-modified silica gel (PSA-2) were observed using scanning electron microscopy (SEM), as shown in Figure 4. The silica gel exhibited a relatively smooth and compact surface with densely packed particles. In contrast, the surface of the amino-modified silica gel appeared rougher, and the particle aggregation became more loosely arranged. This wrinkled and porous surface morphology is beneficial for increasing the number of adsorption sites on the silica gel surface, thereby enhancing its capacity to adsorb pigment molecules [28].

#### 3.1.4. N_2_ Adsorption–Desorption Analysis

**Figure 5:**
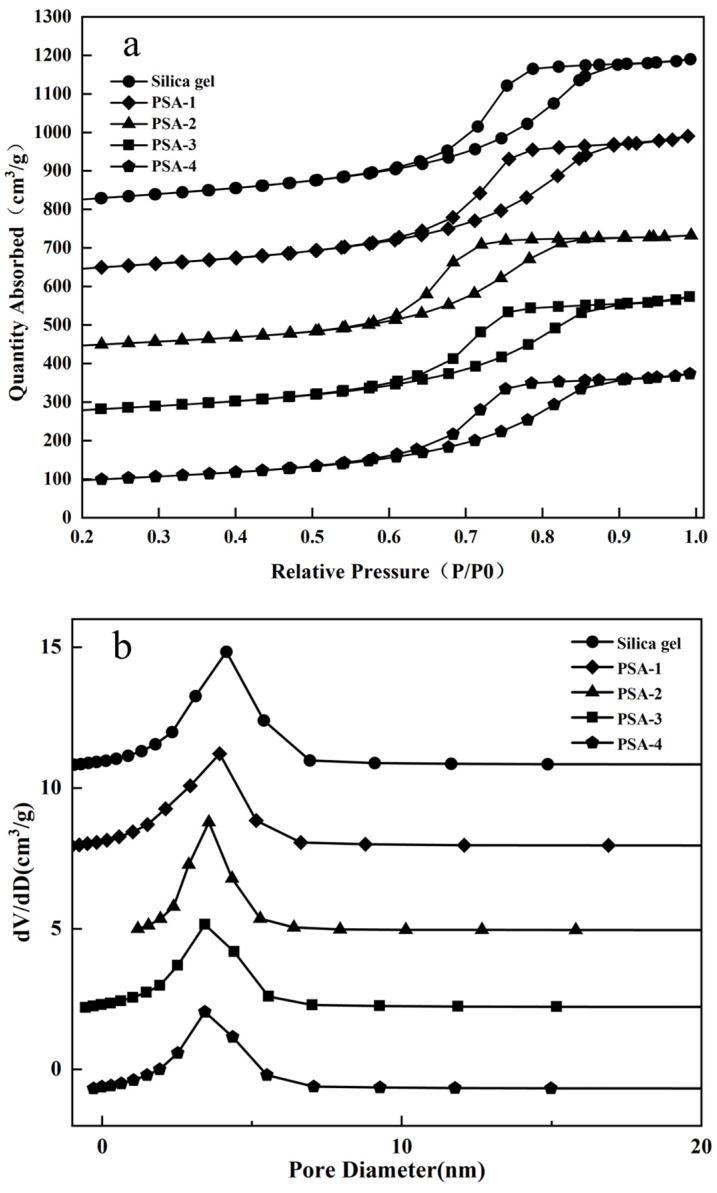
N_2_ adsorption–desorption curves and pore size distribution diagrams of silica gel and amino-modified silica gel: (**a**) N_2_ adsorption–desorption curves; (**b**) pore size distribution diagrams.

Figure 5 shows the N_2_ adsorption–desorption isotherms and pore size distribution curves of silica gel and amino-modified silica gel samples. As illustrated in Figure 5a, both samples exhibited typical type IV isotherms with pronounced H1-type hysteresis loops between the adsorption and desorption branches. These features are characteristic of mesoporous materials, indicating that both silica gel and amino-modified silica gel possessed mesoporous structures with uniform pore distributions [29]. The pore size distribution curves (Figure 5b) further revealed that the pore sizes were relatively narrow and mainly concentrated in the 2–10 nm range. After modification, the pore size of silica gel showed no significant change, indicating that the original mesoporous structure was well preserved. Textural parameters of the samples are summarized in Table 1. With increasing amounts of coupling reagent, both the specific surface area and pore volume of the samples gradually decreased, accompanied by a reduction in average pore diameter. This phenomenon is attributed to the substitution of surface hydroxyl groups on the silica gel by coupling reagent. The grafted silane groups likely penetrated into the pore channels, occupying part of the internal space and thereby reducing the effective pore size [30].

### 3.2. Decolorization Performance of Adsorbents on S. crispa Polysaccharide Solutions

**Figure 6:**
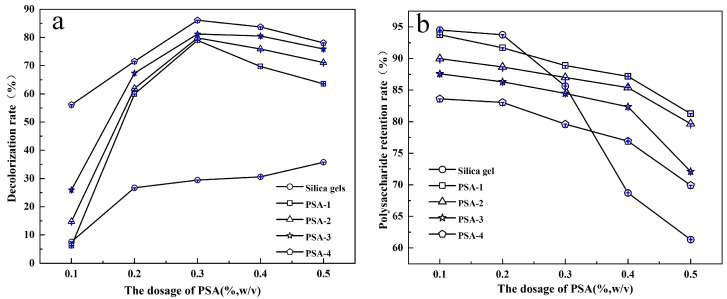
Evaluation of decolorization effects of silica gels and amino-modified silica gels on polysaccharide solution from *S. crispa*: (**a**) decolorization rate and (**b**) polysaccharide retention rate. Note: The purple lines in the figure represent error bars.

Figure 6 illustrates the changes in decolorization rate and polysaccharide retention rate of crude *S. crispa* polysaccharide solutions treated with silica gel-based adsorbents. As shown in Figure 6a, both silica gel and amino-modified silica gel (PSA) exhibited an increasing decolorization rate with the increase in adsorbent dosage. However, the decolorization performance of the PSA was significantly higher than that of silica gel. With increased PSA dosage, the decolorization rate continued to improve, albeit marginally. When the dosage reached 0.3% (*w*/*v*), the decolorization rate peaked at 89%. Figure 6b shows the polysaccharide retention rate after decolorization. The retention rate decreased as the adsorbent dosage increased. The silica gel led to a sharp decline in polysaccharide retention, whereas the PSA resulted in a more gradual reduction. This is primarily because the hydroxyl groups on the surface of silica gel tend to form hydrogen bonding interactions with polysaccharides, leading to the adsorption of polysaccharides on the surface. Since the pigments in crude polysaccharides are mainly phenols and their oxides, which exhibit weak polarity, the surface polarity of silica gel modified with coupling agents is reduced. Thus, both the decolorization rate and polysaccharide retention rate of the modified silica gel increased. Considering both the decolorization rate and polysaccharide retention, the optimal decolorization condition was determined to be 0.15 g of PSA-2, which resulted in a decolorization rate of 79.85% and a polysaccharide retention rate of 86.54%. Decolorization by PSA-2 achieved high pigment removal while maintaining significantly high polysaccharide retention.

### 3.3. Reusability and Regeneration Performance

**Figure 7:**
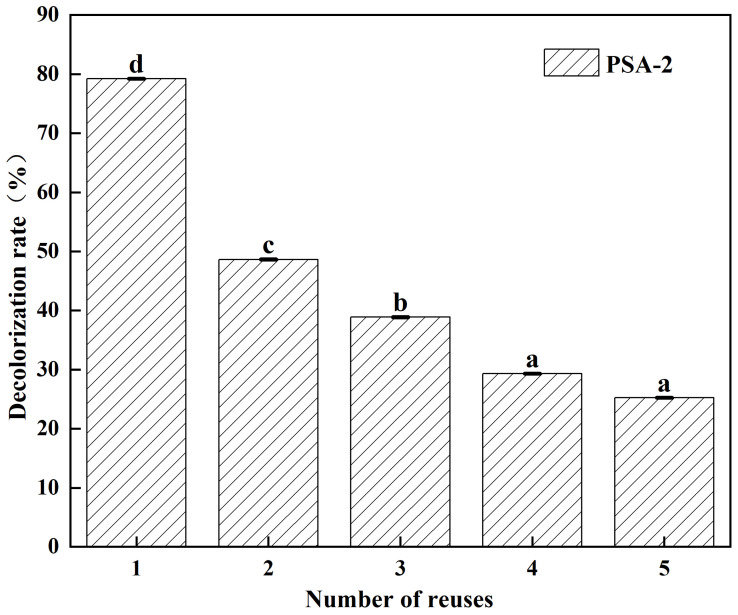
Graph of the reusability of amino-modified silica gels PSA-2. Values with different letters are significantly different (*p* < 0.05).

Given the favorable decolorization efficiency and polysaccharide retention of PSA-2, its reusability and regeneration performance were further evaluated. Figure 7 illustrates the changes in decolorization efficiency of PSA-2 over five consecutive reuse cycles for crude *S. crispa* polysaccharides. The decolorization efficiency of PSA-2 exhibited a decreasing trend with increasing reuse cycles. Notably, a significant reduction (*p* < 0.05) in decolorization rate was observed during the second cycle, with a decline of approximately 41%. However, in subsequent cycles (3rd to 5th), the rate of decline became much less pronounced (*p* < 0.05), with only a 29% drop compared to the first use.

**Figure 8:**
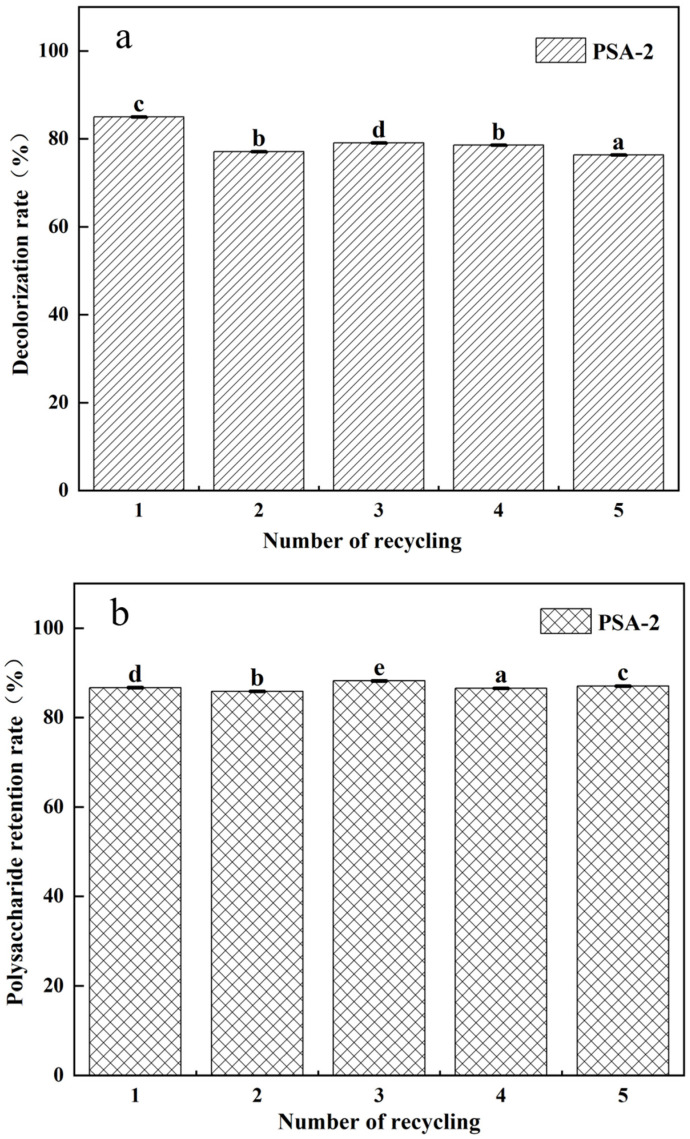
Regeneration performance of amino-modified silica gels PSA-2: (**a**) decolorization rate of *S. crispa* polysaccharides, (**b**) retention rate of *S. crispa* polysaccharides. Values with different letters are significantly different (*p* < 0.05).

Regener ability is a key indicator for the industrial application of decolorizing agents, as it plays a crucial role in reducing operational costs. Figure 8 shows the variations in decolorization rate and polysaccharide retention rate of PSA-2 during five adsorption–desorption regeneration cycles. Throughout the cycles, PSA-2 consistently maintained a high decolorization efficiency (>75%) and polysaccharide retention rate (>80%), indicating its excellent stability and reusability. These results demonstrated that PSA-2 was an effective decolorant for crude *S. crispa* polysaccharides, capable of sustaining favorable performance through simple regeneration procedures.

### 3.4. Adsorption Mechanism of Modified PSA Toward S. crispa Pigments

**Figure 9:**
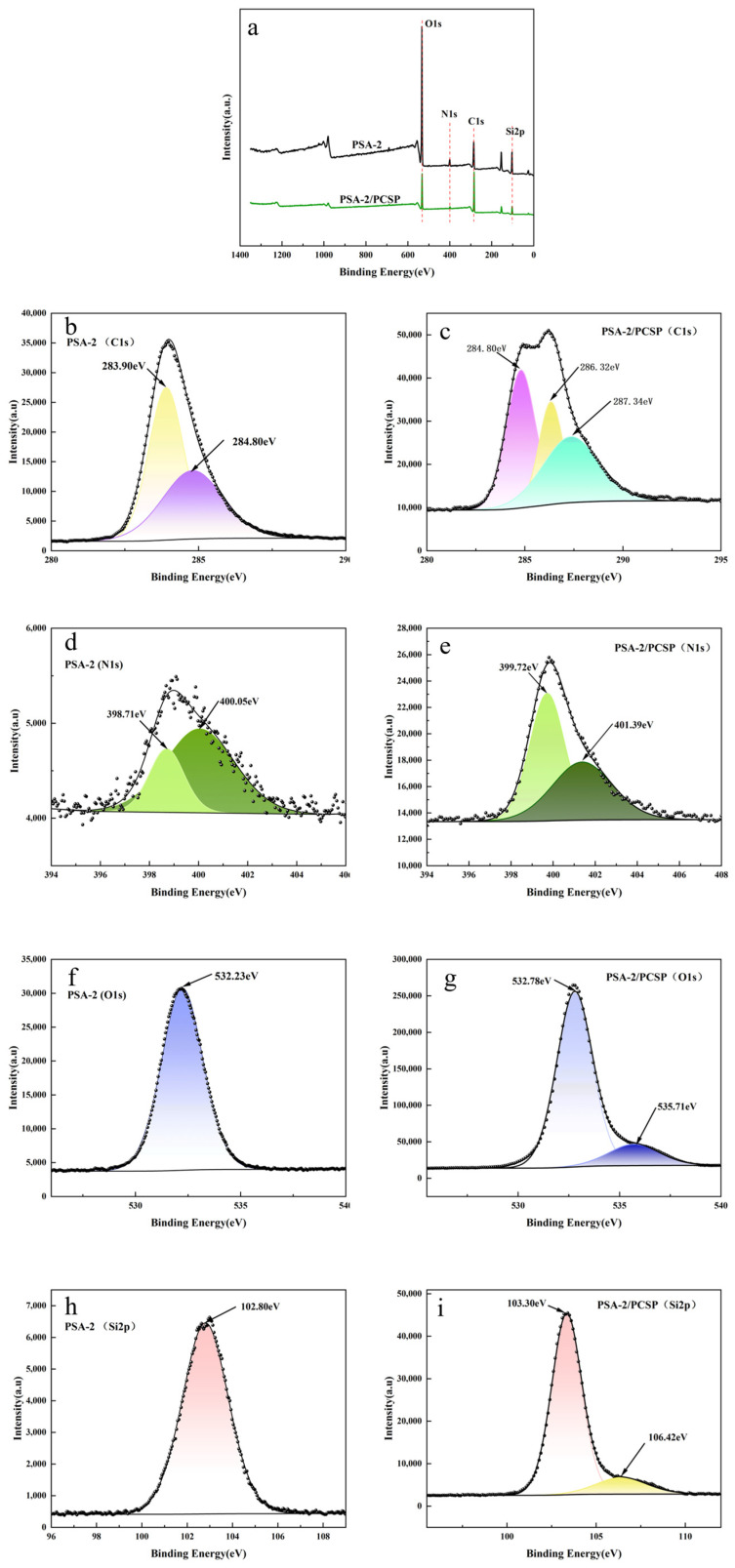
XPS spectra of silica gel and amino-modified silica gel after adsorbing pigments from crude *S. crispa* polysaccharides: (**a**) Full-scan spectra of silica gel and amino-modified silica gel after adsorbing pigments from crude *S. crispa* polysaccharides; (**b**,**c**) XPS spectra of C1s for silica gel and amino-modified silica gel after adsorbing pigments from crude *S. crispa* polysaccharides; (**d**,**e**) XPS spectra of N1s for silica gel and amino-modified silica gel after adsorbing pigments from crude *S. crispa* polysaccharides.; (**f**,**g**) XPS spectra of O1s for silica gel and amino-modified silica gel after adsorbing pigments from crude *S. crispa* polysaccharides; (**h**,**i**) XPS spectra of Si2p for silica gel and amino-modified silica gel after adsorbing pigments from crude *S. crispa* polysaccharides.

To investigate the adsorption mechanism of amino-modified silica gel (PSA-2) toward pigments in crude *S. crispa* polysaccharides (PCSP), X-ray photoelectron spectroscopy (XPS) was employed to analyze the surface elemental composition of PSA-2 before and after adsorption. As shown in the survey spectra in Figure 9a, both samples contained C, N, O, and Si elements. High-resolution XPS spectra of C, N, and O were further analyzed. In the C1s spectrum of PSA-2 Figure 9b, two characteristic peaks were observed at 283.90 eV and 284.80 eV, with the latter representing the graphite carbon reference and the former attributed to Si–C bonds from the coupling reagent. After pigment adsorption, two new peaks appeared at 286.32 eV and 287.34 eV Figure 9c, corresponding to C–O bonds (from polysaccharides and phenolic compounds) and C=O bonds (from oxidized phenolics), respectively. The N1s spectra before and after adsorption Figure 9d,e showed a significant shift in binding energy: from 398.71 eV (–NH_2_) and 400.05 eV (–NH−) to 399.72 eV and 401.39 eV, respectively. These shifts (>1.0 eV) suggest a strong electronic effect, likely driven by hydrogen bonding and π–π conjugation with phenolic hydroxyl groups, which significantly reduce the electron density of nitrogen atoms. In contrast, hydroxyl groups in polysaccharides form weaker hydrogen bonds, insufficient to cause such substantial shifts. These results suggest that phenolic pigments in crude *S. crispa* are adsorbed onto PSA-2 via strong hydrogen bonding interactions with surface amine groups. Moreover, comparison of the O1s and Si2p binding energies before and after adsorption Figure 9f–i revealed increases of approximately 0.5 eV. This may be attributed to extensive hydroxyl groups (-OH) in polysaccharides forming hydrogen bonds with surface silanol groups (Si–OH) on silica. During hydrogen bonding, the lone pair electrons of O in Si-OH shift toward the hydrogen atom in the -OH group of the polysaccharide, decreasing electron density on O and thereby increasing the O1s binding energy. This change in electron density propagates through the Si–O bond, lowering the electron density of Si and raising the Si2p binding energy. Additionally, a new O1s peak at 535.71 eV appeared, which may be attributed to the carbonyl oxygen in uronic acid residues of *S. crispa* polysaccharides. In summary, these XPS results suggest that phenolic pigments may be adsorbed onto PSA-2 via hydrogen bonding with amine groups, while polysaccharide molecules may be involve bound through hydrogen bonding to surface silanol groups on amino-modified silica gel [2,31,32]. Notably, the proposed adsorption mechanism is inferred from XPS derived binding energy shifts, and direct evidence would be required to fully validate these interactions. Future studies will focus on integrating multiple characterization techniques to confirm the exact binding modes.

### 3.5. Biological Activities of S. crispa Polysaccharides

#### 3.5.1. Antioxidant Activity of *S. crispa* Polysaccharides

**Figure 10:**
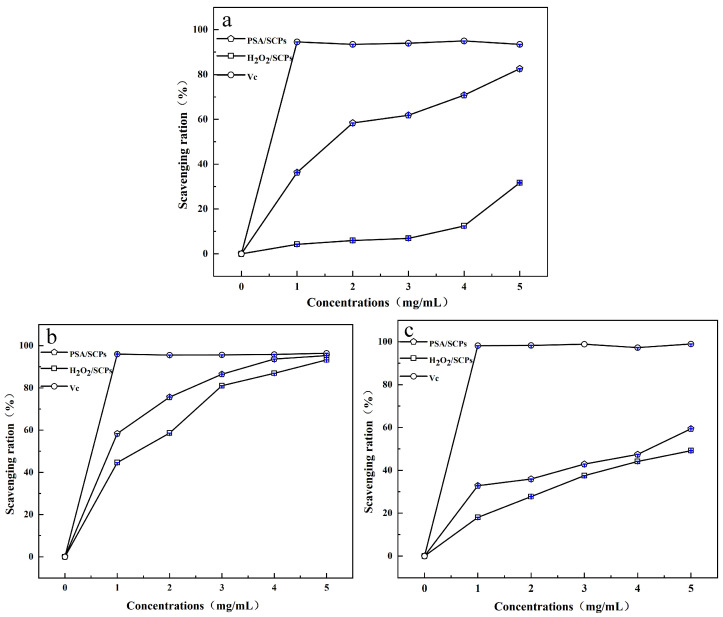
Antioxidant activities of *S. crispa* polysaccharides decolorized by H_2_O_2_ and PSA-2: (**a**) DPPH radical scavenging capacity; (**b**) ABTS radical scavenging capacity; (**c**) hydroxyl radical scavenging capacity. Note: The purple lines in the figure represent error bars.

The antioxidant activities of *S. crispa* polysaccharides were evaluated by measuring their scavenging abilities against DPPH, ABTS, and hydroxyl radicals. The effects of physical decolorization using PSA and chemical decolorization using hydrogen peroxide (H_2_O_2_) were systematically compared, as shown in Figure 10. The polysaccharides decolorized by PSA exhibited excellent antioxidant activity, with the scavenging rates of all three radicals increasing in a dose-dependent manner. At a concentration of 5 mg/mL, the DPPH, ABTS, and hydroxyl radical scavenging rates reached 82.59%, 95.30%, and 59.38%, respectively. In contrast, the polysaccharides treated with H_2_O_2_ showed lower radical scavenging capacities. At a concentration of 0.5 mg/mL, the DPPH, ABTS, and hydroxyl radical scavenging rates were 31.66%, 93.26%, and 49.16%, respectively. The antioxidant activity of *S. crispa* polysaccharides is reportedly closely related to their intact β-(1→3)-glucan backbone and β-(1→6)-linked branches, based on existing literature. As a strong oxidizing agent, H_2_O_2_ is known to potentially break glycosidic bonds in polysaccharide chains, which may disrupt the helical and branched structures and reduce the number of active sites-consistent with the well-documented oxidative degradation behavior of polysaccharides [33,34,35]. As a result, chemical decolorization by H_2_O_2_ is hypothesized to significantly compromise the antioxidant capacity of *S. crispa* polysaccharides, given the established link between structural integrity and the biological activities of β-glucans, though direct evidence from structural (e.g., molecular weight, conformation) and activity assays of polysaccharides pre- and post-H_2_O_2_ treatment is not provided in this study.

#### 3.5.2. Regenerative Effects of *S. crispa* Polysaccharides on Zebrafish Caudal Fin

**Figure 11:**
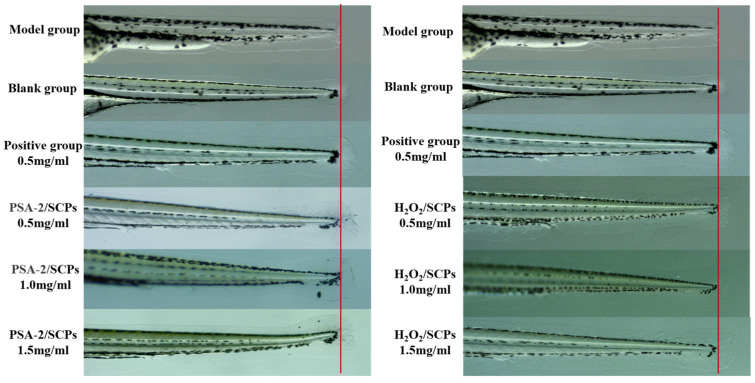
Representative diagram of the fin-repair effect of *S. crispa* pure polysaccharides decolorized by H_2_O_2_ and PSA-2 on zebrafish.

**Figure 12:**
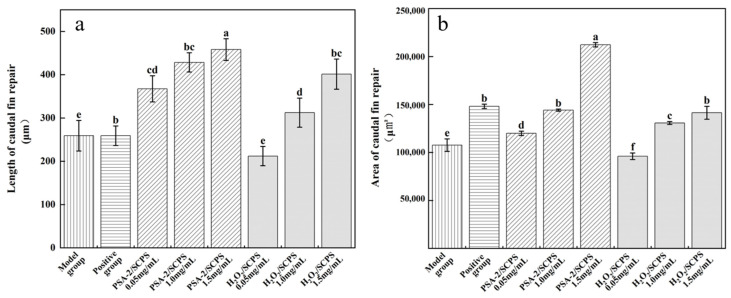
Statistical graph of the caudal fin repair effect of *S. crispa* pure polysaccharides decolorized by H_2_O_2_ and PSA-2 on zebrafish: (**a**) repair length of zebrafish caudal fin; (**b**) repair area of zebrafish caudal fin. Values with different letters are significantly different (*p* < 0.05).

Zebrafish caudal fins are widely used in tissue regeneration studies due to their excellent regenerative capacity and simple anatomical structure, lacking muscle and cartilage tissues. The fin regeneration process can be directly observed and quantitatively assessed by measuring the length and area of fin regrowth [36,37]. Figure 11 presents representative images of caudal fin regeneration in zebrafish treated with *S. crispa* polysaccharides decolorized by PSA-2 and H_2_O_2_. Observations under a 3× microscope revealed that both treatments promoted fin regeneration compared to the control group. Furthermore, the degree of regeneration increased with rising polysaccharide concentration, indicating a dose-dependent repair-promoting effect of *S. crispa* polysaccharides. As shown in Figure 12a, the fin regeneration length in the PSA-2-treated group increased with concentration. At concentrations of 0.5, 1.0, and 1.5 mg/mL, the caudal fin regrowth lengths were 367.46 µm, 428.54 µm, and 458.18 µm, corresponding to increases of 41.82%, 65.40%, and 76.84% compared with the blank control. In contrast, the H_2_O_2_-treated group exhibited lower regeneration lengths of 212.00 µm, 312.50 µm, and 401.32 µm at the same concentrations, with minimal improvement at 0.5 mg/mL and growth rates of only 20.60% and 54.89% at 1.0 and 1.5 mg/mL, respectively. Statistical analysis confirmed that at all tested concentrations, the PSA-2 group promoted significantly greater fin regeneration than the H_2_O_2_ group (*p* < 0.05). In terms of regeneration area, a similar trend was observed, as shown in Figure 12b. The PSA-2-treated group displayed increased fin regrowth areas of 119,957 µm^2^, 144,117 µm^2^, and 212,251 µm^2^ at concentrations of 0.5, 1.0, and 1.5 mg/mL, corresponding to increases of 11.46%, 33.91%, and 97.22%, respectively. In comparison, the H_2_O_2_-treated group exhibited regeneration areas of 98,712 µm^2^, 130,340 µm^2^, and 146,073 µm^2^, with no improvement at 0.5 mg/mL and relative increases of only 21.46% and 31.40% at higher concentrations. Statistical results confirmed that PSA-2-decolorized polysaccharides significantly outperformed H_2_O_2_-treated samples in promoting caudal fin repair (*p* < 0.05). The regenerative effects of polysaccharides are generally attributed to their ability to promote cell proliferation, modulate immune responses, enhance antioxidant defense, and regulate gut microbiota [5,38]. The inferior performance of H_2_O_2_-decolorized polysaccharides may be due to potential oxidative damage to their structural and functional groups during treatment-consistent with the well-documented susceptibility of polysaccharides to oxidative degradation-which may reduce their biological efficacy. This hypothesized structural degradation is further proposed to impair their ability to stimulate cell proliferation, regulate inflammation, and participate in tissue regeneration, based on existing literature linking polysaccharide structural integrity to these bioactive functions. In contrast, PSA-2 decolorization, which relies on physical adsorption rather than chemical oxidation (a process known to induce structural breakdown), is hypothesized to cause minimal structural damage and potentially preserve the bioactive moieties essential for promoting fin regeneration. As a result, PSA-2 maintains higher polysaccharide integrity compared with H_2_O_2_ treatment, which may contribute to improved biological activity. A finding consistent with the working hypothesis that structural preservation supports sustained bioactivity-though direct evidence for structural integrity (e.g., molecular weight distribution, conformational analysis) of polysaccharides post-decolorization with either method is not provided herein.

## 4. Conclusions

In this study, 3-(2-aminoethylamino) propyltriethoxysilane (APS) was used as a modifier to successfully perform amination modification on the hydroxyl groups of silica gel surface, thereby synthesizing a high-efficiency decolorizing agent (PSA) for crude polysaccharides from *S. crispa*. The decolorizing agent consists of mesoporous-structured nanospherical particles. The decolorization rate of PSA-2 for crude *S. crispa* polysaccharides reaches 79.85%, with a polysaccharide retention rate of 86.54%. After five cycles of regeneration and reuse, the decolorization rate for *S. crispa* polysaccharides remains >75%. The pigments in crude *S. crispa* polysaccharides may be adsorbed onto the amino groups of the decolorizing agent. Compared with *S. crispa* polysaccharides decolorized by chemical treatment with H_2_O_2_, polysaccharides decolorized by the PSA exhibit better antioxidant activity and zebrafish caudal fin repair activity. The superior biological activity of PSA-treated polysaccharides may be associated with reduced oxidative degradation, although further structural analysis is needed to confirm this. Therefore, as a novel and high-efficiency decolorizing agent for crude *S. crispa* polysaccharides, PSA may provide a safer alternative for food-grade polysaccharide purification and has promising prospects for practical application.

## Figures and Tables

**Table 1:** The table of pore structure parameters for silica gel and amino-modified silica gels.

Adsorbents	BET Surface Area (Mean ± SEM, m^2^/g)	BJH Desorption Cumulative Volume of Pores (Mean ± SEM, cm^3^/g)	BJH Desorption Average Pore Radius (Mean ± SEM, nm)
Silica gels	320.57 ± 1.7 (*n* = 3)	0.70 ± 0.01 (*n* = 3)	7.15 ± 0.02 (*n* = 3)
PSA-1	287.90 ± 2.2 (*n* = 3)	0.66 ± 0.03 (*n* = 3)	7.15 ± 0.04 (*n* = 3)
PSA-2	222.13 ± 0.9 (*n* = 3)	0.54 ± 0.03 (*n* = 3)	5.86 ± 0.02 (*n* = 3)
PSA-3	230.71 ± 1.1 (*n* = 3)	0.55 ± 0.01 (*n* = 3)	6.78 ± 0.03 (*n* = 3)
PSA-4	216.30 ± 1.8 (*n* = 3)	0.52 ± 0.04 (*n* = 3)	6.83 ± 0.03 (*n* = 3)

## Data Availability

The original contributions presented in this study are included in the article. Further inquiries can be directed to the corresponding author.

## Abbreviations

The following abbreviations are used in this manuscript:

PSA	Primary Secondary Amine
APS	3-(2-aminoethylamino)propyltriethoxysilane
PCSP	Crude *S. crispa* polysaccharides
